# Autism, autistic traits and multiple risk behaviours in adolescence: a longitudinal birth cohort study

**DOI:** 10.1017/S0033291722000940

**Published:** 2023-07

**Authors:** Amanda Ly, Jon Heron, Dheeraj Rai, Caroline Wright

**Affiliations:** 1 MRC Integrative Epidemiology Unit, Population Health Sciences, Bristol Medical School, Bristol, UK; 2 Centre for Academic Mental Health, Population Health Sciences, Bristol Medical School, Bristol, UK; 3 Centre for Public Health, Population Health Sciences, Bristol Medical School, Bristol, UK; 4 BASS Autism Services for Adults, Avon & Wiltshire Partnership NHS Trust, Bristol, UK; 5 NIHR Biomedical Research Centre, Bristol, UK

**Keywords:** Autism, autistic traits, multiple risk behaviours, ALSPAC, epidemiology, longitudinal studies

## Abstract

**Background:**

Multiple risk behaviours (MRBs), typically beginning in adolescence, are associated with increased risk of adverse health and social outcomes. The association between autism and MRBs is little understood.

**Methods:**

Data were from the Avon Longitudinal Study of Parents and Children, an UK-based longitudinal, birth cohort study. Exposures were diagnosed autism and four autistic traits: social communication difficulties, pragmatic language, repetitive behaviours and reduced sociability. Outcomes were participation in up to 14 risk behaviours, including alcohol consumption, smoking, risky sexual behaviours and physical inactivity. Outcome data were collected at ages approximately 12, 14, 16 and 18.

**Results:**

Up to 4300 participants were included in latent basis growth curve analyses with adjustment for confounders. Social communication difficulties were associated with an above average level of MRBs engagement at ~12 years (mean difference *β* 0.26; 95% CI 0.13–0.40), and above average rate of engagement from ages ~12–18 (*β* 0.08; 95% CI 0.02–0.13). Repetitive behaviours were associated with above average levels of engagement in MRBs at ~12 years (*β* 0.24; 95% CI 0.09–0.38). Contrastingly, reduced sociability was associated with a reduced rate of engagement in MRBs from ages ~12–18 (*β* −0.06; 95% CI −0.11 to −0.02). In sex-specific analyses, persisting differences in MRB engagement patterns from ages ~12–18 were observed in males with social communication difficulties and females with reduced sociability temperament.

**Conclusions:**

Having elevated levels of some autistic traits appear to have differentiated effects on MRB engagement patterns. These findings could reflect difficulties fitting in and/or coping mechanisms relating to difficulties with fitting in.

## Background

Adolescence is a period of both individualisation and social integration; it is marked by inadequate problem handling that can result in various risk-taking behaviours (Hurrelmann & Richter, 2006). These different risk behaviours, for example, self-harming, alcohol consumption or illicit drug use, reflect processes of externalising, internalising or evasion. Engagement in multiple risk behaviours (MRBs) carries risk of harm or societal disapproval but in the ‘social ecology of adolescent life’ (Jessor, 1991), this may not be fully comprehended or appreciated by young people. MRBs are associated with increased risk of poor educational attainment and problematic physical and psychosocial outcomes (Busch, Van Stel, Schrijvers, & de Leeuw, 2013; Campbell et al., 2020; Wright, Kipping, Hickman, Campbell, & Heron, 2018). Individuals with autism spectrum disorder (henceforth autism) may find it more difficult than their peers to navigate complex social relations during a time of such flux. Little is known about their engagement in MRBs compared with the general adolescent population.

Though health risk behaviours appear to be patterned by gender, for example, a higher prevalence of cannabis use, anti-social and criminal behaviours in males and a higher prevalence of physical inactivity in females (MacArthur et al., 2012), both engage in a similar number of MRBs rather than having distinct risk profiles (Wright, Heron, Campbell, Hickman, & Kipping, 2020). The MRBs measure comprised a broad range of risk behaviours associated with harm and dimensions of quality of life. It is therefore informative to assess the simultaneous effects of several types of MRBs.

Among the general adolescent population MRBs often co-occur and increase over adolescence (Dumith, Muniz, Tassitano, Hallal, & Menezes, 2012; Hagger-Johnson et al., 2013; Hale & Viner, 2012; Jackson, Sweeting, & Haw, 2012b; Mangerud, Bjerkeset, Holmen, Lydersen, & Indredavik, 2014). There is also evidence that they influence each other in a synergistic manner to increase disease risk, greater than single behaviours alone (Huang, Lanza, Murphy, & Hser, 2012; Plotnikoff et al., 2009). Understanding the factors associated with MRBs is challenging as risk and protective factors may be operating at both individual and aggregate levels. For every incremental decrease in socioeconomic position, the odds of engaging in a greater number of MRBs increases (Kipping, Smith, Heron, Hickman, & Campbell, 2015). Different social mechanisms may be at play, for example, knowledge acquired through education may equip individuals to better understand health-related information, health-related attitudes and behaviours may be influenced by mothers and their level of education (Galobardes, Shaw, Lawlor, Lynch, & Davey Smith, 2006). Regarding MRB prevention, the most promising interventions are complex, multi-component programmes, reflecting the multi-faceted nature of the aetiology of MRBs (Jackson, Henderson, Frank, & Haw, 2012a). This evidence lends further support to public health prevention strategies that focus on reducing MRBs rather than individual risk behaviours in adolescence.

Autism is characterised by difficulties with social communication and social interaction, in addition to repetitive and restricted patterns of behaviours and interests (Lai, Lombardo, & Baron-Cohen, 2014). The prevalence of autism is estimated to be between 1% and 2% in high-income countries (Atladottir et al., 2015; Autism and Developmental Disabilities Monitoring Network Surveillance Year 2008 Principal Investigators, 2012). The autism spectrum is heterogeneous, and individuals may have varying degrees of difficulties across a range of developmental domains. As autism is heritable, genetic liability for the condition can be regarded as a continuum, with diagnosable autism at one extreme end. Consequently, individuals who do not meet current diagnostic criteria may nevertheless have genetic liability for autism and may exhibit recognisable autistic traits. Individuals with autism and autistic traits will vary with respect to ability to cope, mask or compensate with difficulties that may be experienced in daily life.

Individual risk behaviours or small clusters of MRBs are little researched in autism. Whilst no or low prevalence of alcohol use, tobacco smoking nor drug use has been found in Norwegian autistic adolescents (Mangerud et al., 2014), autism was associated with increased risk of substance use-related problems in a Swedish population-based study of predominantly adults (Butwicka et al., 2017). Autistic traits increased the risk of substance use and cannabis use disorders in a cross-sectional study of adult Australian twins (De Alwis et al., 2014). The evidence base on autism and the criminal justice system reflect studies with sample populations that are not representative of all individuals with autism (King & Murphy, 2014). In one large, population-based study, autism diagnosis was linked to lower risk of violent offending, although this was the case after co-morbid attention-deficit/hyperactivity disorder and conduct disorder were adjusted for (Heeramun et al., 2017). To highlight, some individual risk behaviours are inherently social. They may relate to social bonding activities, e.g. alcohol consumption, or social context, e.g. cycling. Some individual risk behaviours are not e.g. self-harm and in some cases physical inactivity and excessive television watching. However, we do not know how multiplicity of risk behaviours may manifest in autistic adolescents or those with autistic traits. Engagement in MRBs among autistic individuals may also differ between adolescence and adulthood. Previous findings could also be due to selective samples of participants and inadequate adjustment for confounding which would bias effect estimates. Large, population-based studies with longitudinal data are needed to be more confident in the temporality of associations and external validity of study results.

Such studies may provide insights into whether adolescents with autism or autistic traits are at higher risk of engagement in MRBs. Modelling MRB trajectories in studies with repeated measures, in other words estimating rates of change over time, may also enable us to identify critical periods amenable to intervention.

The objectives of this study were to investigate (1) whether autism and autistic traits were associated with engagement in MRBs from ~12–18 years of age; (2) whether the associations differed by sex.

## Methods

### Study design, setting and participants

Participants were the index children from the Avon Longitudinal Study of Parents and Children (ALSPAC) birth cohort study (Fraser et al., 2012). Briefly, 14 541 pregnant women with expected delivery dates between April 1991 and December 1992 from Bristol and the surrounding areas, UK, were initially recruited. Out of the 13 988 children part of the original sample, who were alive at 1 year of age, data from singletons or first-born twins were selected. In the final analytic samples, up to 4300 participants were included. A diverse range of data has been and continues to be collected on a frequent basis, including data on individual risk behaviours, lifestyle and socio-demographic characteristics (Boyd et al., 2013). Please note that the study website contains details of data that are available through a fully searchable data dictionary and variable search tool (www.bris.ac.uk/alspac/researchers/data-access/data-dictionary). The ALSPAC Ethics and Law Committee and the local research ethics committees provided the ALSPAC study with ethical approval to collect all data.

### Exposures

Diagnosed autism (case = 1; non-case = 0) in participants was identified using a multisource approach. This was through reviewing the educational records of children requiring special needs support, parents reporting a given clinical diagnosis of autism or Asperger Syndrome, and clinical record review for children identified as having a developmental disorder. These cases were adjudicated by a consultant paediatrician using the *International Statistical Classification of Disease*, 10th revision. These autism cases were also cross-validated against autism trait measures (Golding et al., 2017; Guyatt, Heron, Knight, Golding, & Rai, 2015), and were found to be associated with a polygenic risk score of autism, a measure of common genetic variation that is associated with autism (Rai et al., 2018). Those with intellectual disability (ID) were included.

ALSPAC collected 93 measures related to autistic features up to 11 years of age. Of these, 88 were derived from self-completed questionnaires, with the remainder from carer-completed questionnaires. Four individual measures, described as traits here, were the strongest predictors of autism (Steer, Golding, & Bolton, 2010). Initially, we took continuous scales representing these four autistic traits: sociability as part of the Emotionality, Activity and Sociability Temperament scale (assessed at 38 months); repetitive behaviours (assessed at 69 months); pragmatic language difficulties from the Children's Communication Checklist (assessed at 115 months) and social communication difficulties as measured by the Social Communications Disorder Checklist (SCDC, assessed at 91 months). To select participants experiencing the highest levels of difficulties, we reversed the scale and selected cut-offs to loosely represent the top ‘most affected’ decile of each trait distribution (Guyatt et al., 2015) (i.e. worst 10% = 1, remaining 90% not affected or less affected = 0.)

### Outcomes

We created continuous MRB scores at four timepoints (approximately 12, 14, 16 and 18 years of age) across adolescence, reflecting engagement in up to 14 MRBs. These were derived from child participants' responses to postal questionnaires and questions asked during clinical visits. Individual risk behaviours are detailed in Table 1. MRBs are age-calibrated and amended to distinguish between normative and risky behaviours. For example, any alcohol consumption at 12 years is considered a risk behaviour but hazardous alcohol consumption is a risk behaviour at 18 years.

**Table 1.** Individual risk behaviours assessed at each timepoint

~Age 12 (mean age 10.65 years at F10, mean age 11.75 years at F11^a^)	
Anti-social behaviour	Reported that they had engaged in anti-social behaviour
Smoking	Has ever smoked
Alcohol consumption	Has ever consumed alcohol
Cannabis use	Has used cannabis
Physical inactivity	Has typically over the past year exercised <5 times per week
Self-harm	Has purposely hurt themselves in some way in their lifetime
Risky sedentary behaviour	Watches 3+ hours of TV every day on average
Unhealthy diet	Does not maintain a balanced diet
~Age 14 (mean age 14.04 years at CCQ, mean age 13.84 at TF2)	
Anti-social behaviour	Reported that they had engaged in anti-social behaviour
Smoking	Has ever smoked
Alcohol consumption	Has drank 2+ whole drinks in the last 6 months
Physical inactivity	Has typically over the past year exercised <5 times per week
Risky sedentary behaviour	Watches 3+ hours of TV every day on average
Unprotected sex	Has had sexual intercourse without contraception
Risky sexual behaviour	Has had sexual intercourse with 3 or more young people
Cannabis use	Has used cannabis in the last 6 months
Illicit drug/solvent use	Has tried other drugs to get high
Unhealthy diet	Does not maintain a balanced diet
~Age 16 (mean age 15.47 years at TF3, mean age 16.68 years at CCS)	
Car passenger risk	Has taken a passenger risk at least once in their life
Scooter risk	Has driven a motorbike/scooter off road, or without a licence on a public road
Cycle helmet use	Had last ridden a bicycle without having worn a helmet on a public road
Illicit drug/solvent use	Had either been a regular user (i.e. used 5+ times) of 1+ illicit drug
Cannabis use	Reported sometimes using cannabis
Regular tobacco use	Has ever smoked and is regularly smoking by currently smoking at least one cigarette
Hazardous alcohol consumption	In the past year has scored 8+ on the Alcohol Use Disorders Identification list (AUDIT)
Unprotected sex	No contraception on the last occasion they had sexual intercourse
Risky sexual behaviour	Has had sexual intercourse with 3+ young people in the last year
Self-harm	Has purposely hurt themselves in some way in their lifetime
Criminal/anti-social behaviour	Reported that at least once in the past year they had undertaken at least one anti-social or criminal behaviour
Risky sedentary behaviour	Spent 3+ hours watching television on average per day across the week
Physical inactivity	Has typically over the past year exercised <5 times per week
~Age 18 (mean age at 17.82 at TF4, mean age 18.65 years at CCT)	
Cannabis use	Uses cannabis at least once a month
Regular tobacco smoking	Has ever smoked and is regularly smoking
Hazardous alcohol consumption	In the past year has scored 8+ on the AUDIT
Criminal/anti-social behaviour	Has undertaken at least 1 anti-social or criminal behaviour 1+ in the past year
Illicit drug/solvent use	Has used any drug/solvent/inhalant over the past year
Unprotected sex	Did not use condom on most recent time had sex
Risky sexual behaviour	Has had 3+ sexual partners in the past year
Self-harm	Has purposely hurt themselves in some way
Car passenger risk	Has taken a passenger risk at least once in their life
Car passenger risk	Committed at least one risk as driver w/o licence
Scooter risk	Has driven a motorbike/scooter off road, or without a licence on a public road
Gambling	Used at least 1 form of gambling on at least weekly basis
Physical inactivity	Has typically over the past year exercised <5 times per week.

a F- and TF- indicate data collection in focus clinics and CC- via questionnaires

### Covariates

Covariates were selected on an *a priori* basis, based on previous research into MRBs (Campbell et al., 2020; Wright et al., 2018). Primarily, socioeconomic factors have a well-established effect on health outcomes and should be adjusted for, including in relation to MRBs research carried out using ALSPAC data (Kipping et al., 2015). As per previous work, we have also adjusted for some maternal psychopathology and risk behaviours occurring in the offspring's earlier life and offspring ID status. These data were collected at baseline through carer-completed questionnaires, unless otherwise specified. These covariates were: parental occupation, home ownership status, household income and maternal education level, the child's sex and mother's age at delivery. Data on maternal psychopathology, individual maternal risk behaviours and financial difficulties via self-completed questionnaires were collected at 38 months. We created a binary variable for cases of ID, taking IQ measured by the Wechsler Intelligence Scale for Children at age 8 and dichotomising it to <70 *v.* ≥70.

### Statistical analysis

We calculated means with standard deviations of continuous variables as well as frequencies of binary and ordered categorical variables for participants with at least one MRB score at any and up to four timepoints.

**Fig. 1.**
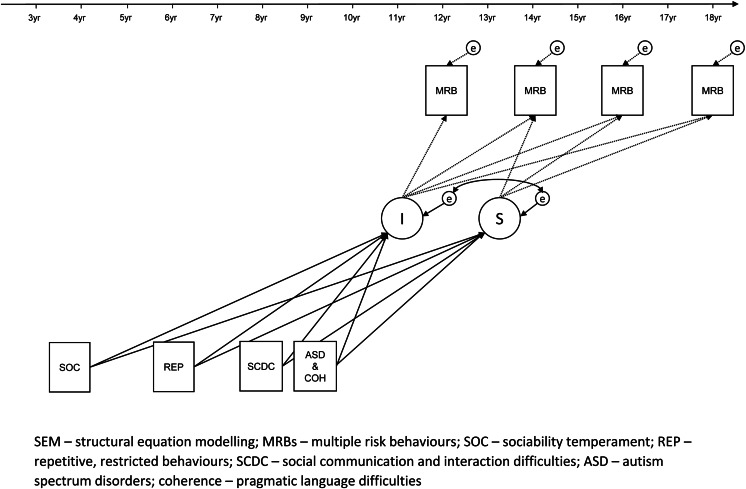
SEM path diagram illustrating the investigation of autism-related exposures and MRBs in adolescence (covariates not shown for simplicity).

As preliminary analyses, we used multivariable regression analysis to explore associations between autism-related exposures and MRB scores at each timepoint. Trajectories were non-linear and mostly downward except for social communication difficulties where the trajectory was upward (online Supplementary Table S1). Upward and downward trajectories indicate increases and decreases in the number of MRBs respectively, as time progressed. We then used two multilevel Structural Equation Modelling (SEM) techniques to model MRB growth curves (trajectories). The SEM framework enables use of the full information maximum likelihood (FIML) method; participants who have at least one MRB score and more complete data on exposures and covariates could be included. We used latent basis growth curve modelling as it produced the best-fitting models (online Supplementary Table S2). These models are illustrated in Fig. 1.

We initially fitted growth curve models without autism-related exposures to estimate overall average engagement in MRBs. Following that, we estimated the associations between autism, autistic traits and average engagement in MRBs at ~12 years of age and the rate of change from ~12 to ~18 years of age, by fitting growth curve models with no covariates. We then fitted models adjusted for sex, and all other covariates. We also tested whether the associations were moderated by sex. Sex-specific analyses were performed as this has been done previously (Wright et al., 2020). Analyses were performed in Stata version 15.1 (StatCorp, 2017).

### Missing data

We assessed the differences in sociodemographic characteristics of groups of differing levels of missingness (online Supplementary Tables S3 and S4). We also used the default listwise deletion maximum likelihood method, including participants with MRB scores from all four timepoints for a complete case analysis (online Supplementary Table S5). We also calculated the proportion of complete cases as participants who were in adjusted complete cases analyses over participants who were in adjusted FIML analyses.

### Additional analyses

We have also performed additional analysis for contextualisation. Firstly, we computed distributions of the exposures by sociodemographic characteristics and other variables for participants with at least one MRB score. Secondly, we calculated distributions for individual risk factors by exposures at ~16 years of age. We chose 16 as there is a body of epidemiological research into MRBs at this age, suggesting it is a possible period of intervention. Participants included were those who were included in latent basis growth curve modelling, adjusted for all confounding variables. χ^2^ tests were also performed for hypothesis generating purposes.

## Results

Information on participants with at least one MRB score is presented in Table 2. Just over half were females, 1.09% had autism, 8.21% had social communication and interaction difficulties, 8.94% had pragmatic language issues, 6.11% had repetitive behaviours and 10.8% had reduced sociability. Maternal MRBs engagement was generally very low. Over half of mothers included in analyses had educational qualifications above O-levels. The average number of MRBs participated in at ~12 years was 1.69 (s.d. 1.11), at ~14 years it was 1.89 (1.17), at ~16 years it was 2.91 (1.86) and at ~18 years it was 3.03 (1.97).

**Table 2.** Sociodemographic characteristics for participants with at least one measurement of MRBs

	At least one measurement of MRBs, *n* (%)
*N*	5528
Sex	
Male	2556 (46.24)
Autism cases	
Yes	60 (1.09)
Social communication and interaction difficulties (SCDC)	
Yes	400 (8.21)
Pragmatic language difficulties	
Yes	443 (8.94)
Repetitive behaviours	
Yes	298 (6.11)
Reduced sociability	
Yes	553 (10.80)
Mum had anxiety or nerves^a^	
Yes	955 (18.96)
Maternal cannabis use^a^	
Yes	175 (3.47)
Frequency mum has taken amphetamines, heroin, methadone, crack or cocaine^a^	
Yes	31 (0.62)
Whether mum had major financial problems^a^	
Yes	680 (13.57)
No. of cigarettes mother has smoked each day^a^	
None	4131 (84.05)
<10	309 (6.29)
10–19	329 (6.69)
20+	146 (2.97)
Quantity of alcohol mum drinks^a^	
Don't drink	486 (9.69)
<Once a week	2240 (44.68)
>Once a week	1738 (34.67)
1–2 glasses everyday	504 (10.05)
3–9 glasses everyday	45 (0.90)
Type of property	
Mortgaged/own	4576 (84.99)
Privately rent	403 (7.49)
Sub rent	405 (7.52)
Household income	
High	1163 (25.21)
Middle high	1054 (22.84)
Middle	942 (20.42)
Middle low	829 (17.97)
Low	626 (13.57)
Mother's educational level	
<O-level	1037 (19.27)
O-level	1881 (34.95)
>O-level	2464 (45.78)
Parents’ social class	
Professional	860 (18.03)
Managerial and technical	2197 (46.07)
Skilled non-manual	1128 (23.65)
Skilled manual, part or unskilled	584 (12.25)
	Mean (s.d.**)**
IQ	105.54 (16.03)
Maternal age	29.69 (4.51)
Maternal depression score^a^	6.51 (4.82)
MRBs total score at 12	1.69 (1.11)
MRBs total score at 14	1.89 (1.17)
MRBs total score at 16	2.91 (1.86)
MRBs total score at 18	3.03 (1.97)

MRBs, multiple risk behaviours; *N*, total number in sample; *n* (%), number and percentage in each category; IQ, Intelligence Quotient as measured by the Wechsler Intelligence Scale for Children; Maternal depression score as measured by the Edinburgh Postnatal Depression Scale.

a Since the study child was 18 months old.

The average number of MRBs engaged in at ~12 years was 1.69 (95% CI 1.66–1.72) with an average uptake of 0.26 (95% CI 0.25–0.28) additional MRBs for each passing year up to ~18 years. Results to assess the effects of autism-related exposures on MRBs engagement are presented in Table 3; the comparison groups were: autism cases *v.* non-cases and participants with the highest levels of difficulties experienced and recognised as an autistic trait *v.* those with no or lower levels of difficulties, acknowledged as typically developing here. In models adjusted for all covariates, social communication difficulties were associated with both greater than average MRBs at ~12 years (mean difference *β* 0.26; 95% CI 0.13–0.40) and a marginally faster than average increased uptake of MRBs per year across adolescence (*β* 0.08; 95% CI 0.02–0.13). Repetitive behaviours were also associated with above average number of MRBs at ~12 years (*β* 0.24; 95% CI 0.09–0.38). In contrast, reduced sociability was associated with an uptake of fewer than average number of MRBs per year across adolescence (*β* −0.06; 95% CI −0.11 to −0.02). There was little evidence for an association between diagnosed autism, pragmatic language difficulties and MRBs engagement in adolescence.

**Table 3.** The effects of autism-related exposures on MRBs from ages ~12 to ~18 in ALSPAC participants using the FIML method

Exposure	Effect of exposure on baseline MRB	*p*-value	Effect of exposure on rate of change	*p*-value
	*β* (95% CI)		*β* (95% CI)	
Autism cases				
*N* *=* 4300				
Unadjusted	0.16 (−0.14 to 0.47)	0.30	−0.12 (−0.25 to −0.01)	0.07
Adjusted 1	0.18 (−0.13 to 0.49)	0.26	−0.13 (−0.26 to −0.01)	0.04
Adjusted 2	0.20 (−0.11 to 0.50)	0.21	−0.12 (−0.25 to −0.01)	0.07
SCDC				
*N* *=* 3978				
Unadjusted	0.30 (0.17–0.43)	<0.001	0.08 (0.03–0.14)	0.004
Adjusted 1	0.31 (0.18–0.44)	<0.001	0.08 (0.02–0.14)	0.01
Adjusted 2	0.26 (0.13–0.40)	<0.001	0.08 (0.02–0.13)	0.01
Pragmatic language difficulties				
*N* *=* 3991				
Unadjusted	0.12 (−0.004 to 0.25)	0.06	−0.01 (−0.05 to 0.06)	0.78
Adjusted 1	0.13 (0.002–0.25)	0.05	0.003 (−0.05 to 0.06)	0.95
Adjusted 2	0.07 (−0.06 to 0.19)	0.28	−0.002 (−0.06 to 0.05)	0.93
Repetitive behaviours				
*N* *=* 3997				
Unadjusted	0.26 (0.11–0.41)	0.001	−0.01 (−0.08 to 0.05)	0.65
Adjusted 1	0.27 (0.12–0.42)	0.001	−0.02 (−0.08 to 0.04)	0.51
Adjusted 2	0.24 (0.09–0.38)	0.002	−0.02 (−0.09 to 0.04)	0.45
Reduced sociability				
*N* *=* 4204				
Unadjusted	0.06 (−0.04 to 0.17)	0.24	−0.06 (−0.11 to −0.02)	0.005
Adjusted 1	0.07 (−0.04 to 0.18)	0.21	−0.07 (−0.11 to −0.02)	0.003
Adjusted 2	0.06 (−0.05 to 0.16)	0.28	−0.06 (−0.11 to −0.02)	0.004

*β* (95% CI) – beta coefficient representing a mean different point estimate; CI representing confidence intervals; SCDC, social communication and interaction difficulties as measured by the Social Communication Disorder Checklist

Adjusted 1: adjusting for participant’ sex only.

Adjusted 2: for all covariates, including postnatal anxiety, maternal cannabis since study child was 18 months old, amphetamines use since study child was 18 months old, maternal use of heroin, methadone, crack or cocaine since the child was 18 months old, postnatal depression, major financial difficulties since the study child was 18 months old, maternal smoking, maternal alcohol consumption, maternal age, type of tenure, household income, maternal educational level, parental social class, IQ and participant's sex

Results from sex-specific analysis are presented in Table 4. In models with no autism-related exposures, females engaged in a marginally higher mean number of MRBs than males at ~12 years (females: *β* 1.72; 95% CI 1.68–1.76 *v.* males: *β* 1.65; 95% CI 1.61–1.70). On average, males engaged in MRBs at a faster rate over adolescence compared to females in the ALSPAC study. However, the average male with autism or one of the autistic traits compared to the average female with these difficulties did not differ in their MRBs patterns when compared to each other. There was good evidence to indicate that both males and females with social communication difficulties and repetitive behaviours differed in their MRBs engagement at ~12 years of age when compared to their typically developing counterparts. We also observed differences in MRB engagement patterns in males with social communication difficulties with an above average rate of uptake of MRB over adolescence (*β* 0.11; 95% CI 0.02–0.19). Females with reduced sociability engaged in a below average uptake of MRBs over adolescence (*β* −0.07; 95% CI −0.13 to 0.01).

**Table 4.** The effects of autism-related exposures on MRB from ~12 to ~18 years in ALSPAC participants using the FIML method, stratified by sex, adjusted

	Males	*p*-value	Females	*p*-value	Heterogeneity test
	*β* (95% CI)		*β* (95% CI)		*p*-value
Autism cases % (*n*/*N*)	2.11 (42/1992)		0.56 (13/2308)		
Effect of exposure on baseline MRB	0.29 (−0.07 to 0.65)	0.11	−0.10 (−0.71 to 0.51)	0.75	0.28
Effect of exposure on rate of change	−0.11 (0.26–0.04)	0.16	−0.11 (−0.36 to 0.14)	0.38	0.99
SCDC % (*n*/*N*)	11.13 (208/1868)		5.73 (121/2110)		
Effect of exposure on baseline MRB	0.29 (0.11–0.46)	0.001	0.22 (0.03–0.42)	0.03	0.64
Effect of exposure on rate of change	0.11 (0.02–0.19)	0.01	0.04 (−0.05 to 0.12)	0.38	0.24
Pragmatic language difficulties % (*n*/*N*)	11.36 (211/1857)		5.95 (127/2134)		
Effect of exposure on baseline MRB	0.12 (−0.04 to 0.28)	0.28	0.01 (−0.21 to 0.19)	0.92	0.32
Effect of exposure on rate of change	0.00 (−0.08 to 0.08)	1.00	−0.01 (−0.10 to 0.07)	0.80	0.86
Repetitive behaviours % (*n*/*N*)	7.63 (142/1862)		4.40 (94/2135)		
Effect of exposure on baseline MRB	0.23 (0.03–0.43)	0.02	0.24 (0.02–0.47)	0.04	0.94
Effect of exposure on rate of change	−0.07 (−0.16 to 0.02)	0.12	0.03 (−0.06 to 0.12)	0.54	0.13
Sociability % (*n*/*N*)	12.69 (248/1964)		9.56 (215/2250)		
Effect of exposure on baseline MRB	0.07 (−0.08 to 0.22)	0.37	0.05 (−0.10 to 0.20)	0.50	0.87
Effect of exposure on rate of change	0.06 (−0.13 to 0.01)	0.07	−0.07 (−0.13 to −0.01)	0.02	0.88

In an assessment of missing data, we generally found expected patterns such as socioeconomic differences. Further details in the online Supplementary material include results in Tables S3 and S4. With regards to proportion of complete cases, they were the following: autism 14.1% (606/4300); social communication difficulties 14.7% (585/3978); pragmatic language issues 14.9% (594/3991); repetitive behaviours 14.7% (588/3997) and reduced sociability 14.3% (602/4204).

Cross-tabulations for sociodemographic characteristics and other variables by exposures of interest are presented in online Supplementary Table S6. The key finding was that there are small numbers for analysis with autism diagnosis as an exposure. In online Supplementary Table S7, we present results from cross-tabulations for our exposures and individual risk behaviours at ~16 years of age.

## Discussion

In this population-based cohort study, we assessed the associations between diagnosed autism, autistic traits and engagement in MRBs during adolescence. We found that participants with high levels of social communication difficulties and repetitive behaviours were both associated with an above average number of MRBs at ~12 years, compared to lower levels or no issues with these traits. Additionally, social communication difficulties were associated with an above average rate of uptake between ~12 and ~18 years of age, whilst reduced sociability was associated with a below average uptake of MRBs during the same time frame.

Our results reiterate the importance of studying a broad range of MRBs as an outcome (Wright et al., 2020). They are also consistent with previous studies in that they report differentiated effects of diagnosable autism and autistic traits and outcomes in adolescence and adulthood. We did not find that diagnosed autism was associated with any outcomes, perhaps due to a relatively small number of individuals with autism and therefore results are tentative. Previous studies report that diagnosed autism is associated with decreased risk of committing violent crimes after adjusting for co-morbidities (Heeramun et al., 2017), whilst autistic traits have been associated with increased risk of substance use and misuse, with the except of alcohol consumption (De Alwis et al., 2014), and cyber-crimes (Payne et al., 2019). There is evidence that those with autism and autistic traits have a difficult time in childhood and adolescence and it is possible that MRBs may help them cope. Social communication difficulties in particular are strongly associated with depression and suicidality (Rai et al., 2018). Autistic adolescents or those with autistic traits may have increased susceptibility to mental health issues (Lundstrom et al., 2011; Rai et al., 2018), bully victimisation (Cappadocia, Weiss, & Pepler, 2012; Forrest, Kroeger, & Stroope, 2020; Rai et al., 2018; Zablotsky, Bradshaw, Anderson, & Law, 2014) and bully victim perpetration (Hwang, Kim, Koh, & Leventhal, 2018; Maiano, Normand, Salvas, Moullec, & Aime, 2016). Our study also suggests differing effects of different autistic traits. Explanations for less engagement in some risk behaviours, perhaps applicable to adolescents with reduced sociability, could be attributed to limited social relationships, mis- or poor communications between autistic and non-autistic individuals, reluctance to ‘lose control’ and dislike of being in substance use settings. An explanation for more engagement in risk behaviours is some individuals with social communication difficulties may be having high motivation for socialising and making friends. To cope with difficulties, these individuals may try many types of strategies to self-soothe, and once certain behaviours help, they might be engaged in more excessively. Autistic traits could be interpreted as markers of neurodevelopmental traits as social communication and interaction difficulties can also be seen in other conditions such as attention deficiency hyperactivity disorder (ADHD) or psychosis (Sasson & Bottema-Beutel, 2021). An alternative explanation is that autistic traits are identifying participants who are autistic but may go on to be diagnosed later in adolescence or adult life.

Other findings of interest relate to sex-specific differences. The average male and the average female with autism or one of the autistic traits did not differ from each other with respect to MRB engagement. However, we observed some differences between them from typically developing counterparts. There was good evidence to indicate that both males and females most affected by social communication difficulties and repetitive behaviours differed in their MRBs engagement at ~12 years. Persisting differences in MRBs engagement throughout adolescence for males with social communication difficulties and females with very reduced social temperament perhaps reflect persisting differences that may make it more difficult for these individuals to fit in. This may be consistent with the idea that many autistic females are more proficient at social camouflaging (Hull et al., 2019), that is, performing in a socially acceptable way to seem ‘less autistic’ (Lai et al., 2011). Camouflaging may improve chances of ‘fitting in’ and making friends (Hull et al., 2017). We note that social temperament was measured in early childhood.

Our study has several strengths; we had repeated measures of a diverse selection of risk behaviours and thus were able to study them longitudinally, reducing risk of reverse causation. The study was carried out within a population-based cohort, which generally improves external validity of study results compared to a clinic-based cohort. Data collection was prospective, enabling us to establish temporality of associations and mitigate recall bias. We used the FIML approach which is an alternative approach to handling missing data that is pragmatic and can be more efficient in producing unbiased estimates than multiple imputation under missing at random and missing completely at random missingness mechanisms (Enders & Bandalos, 2001). This is because with multiple imputation, we are modelling uncertainty but with FIML, which is typically used in a SEM framework and assumes multivariate normality, we work with greater completeness in of data on exposures and covariates collected early on in a cohort study but some missingness in the outcome due to attrition (Carpenter & Smuk, 2021). A complete case analysis with adjustment for confounders would substantially reduce the sample size to *N* < 610, resulting in loss of statistical power to detect differences in effects. We also selected multiple autism-related exposures: autism as the combined effect of core and additional autistic traits that is sufficient to warrant a diagnosis, and individual traits. The number of autism cases, despite thorough case finding and adjudication in ALSPAC, was not substantial and thus will affect the precision of their estimated effects. A greater number of participants with elevated levels of autistic traits gave us greater statistical power to investigate their effects.

The limitations of our study should also be acknowledged. Bias due to attrition in cohort studies is inevitable; non-random attrition occurs in the ALSPAC study (Taylor et al., 2018). In our study, we found that adolescents with at least one MRB score at any given timepoint were of higher socioeconomic status (SES) compared to those with no recorded MRBs scores, which is also consistent with previous findings of greater attrition in those of lower SES in cohort studies (Matthews, Chatfield, Brayne, Medical Research Council Cognitive, & Ageing, 2006; Tolonen, Dobson, & Kulathinal, 2005). We did not assess the combined effects of autism/autistic traits and comorbid psychiatric or neurodevelopmental traits and did not assess them as psychiatric conditions in adolescence as mediators; this was beyond the scope of our study. We excluded intellectual disabilities as an effect modifier and confounding factors relating to fathers as our analytic sample size would be reduced to a large degree. Risk behaviours included in MRBs scores were reduced to binary variables, losing some sensitivity in measurement, but this should not make a perceptible difference in population-level average number of MRBs. We also gave equal weighting to each risk behaviour in our MRBs scores and did not consider a different or smaller selection of MRBs.

Our study reiterates the importance of studying the effects of autistic traits, particularly social communication difficulties in future studies. It may also be pertinent to study sex-specific differences as we found some nuanced differences. Emerging evidence suggests that some autistic individuals do not fall into the confines of binary gender identification and thus, such identities should be accommodated if data are available (Warrier et al., 2020). Replication studies in cohorts of comparable or larger sample sizes and with less social stratification are recommended, particularly those with larger numbers of autism cases, to further assess whether autism is associated with increased or decreased risk of MRBs.

Although we have not described them as neurodevelopmental traits, knowing that some autistic traits may increase risk of engaging in MRBs in adolescence is helpful to establish with potential public health implications. Those with such traits could also arguably be undiagnosed autism cases. If it is known that those with social communication and interaction difficulties are at higher risk of MRBs during a period when adolescents are in school and therefore supervised, then this could be a good time for interventions on risk-taking behaviours. This reinforces the notion that public health strategies that aim to reduce MRBs rather than individual risk behaviours in adolescence are promising and that a population-wide approach may also benefit a greater number of individuals that may not be classed as vulnerable or ‘high risk’ in clinical terms.

In summary, adolescents with social communication difficulties were likely to engage in a greater than average number of MRBs across adolescence, which could increase their risk of poor health and social outcomes. We also found that adolescents with repetitive behaviours engaged in above average levels of MRBs at ~12 years. Differing MRB engagement patterns persisted throughout adolescence for males with social communication difficulties and females with reduced sociability. These groups may find it more difficult to fit in and the ‘social ecology of adolescent life’ more difficult to navigate.

## Supplementary material

For supplementary material accompanying this paper visit https://doi.org/10.1017/S0033291722000940. click here to view supplementary material
