# Supplementary material for: Autism, autistic traits and multiple risk behaviours in adolescence: a longitudinal birth cohort study
Source: Psychol Med. 2022 Apr 28;53(9):4210–9. doi: 10.1017/S0033291722000940 (PMC10317793; doi:10.1017/S0033291722000940)
Supplement: Supplementary file 1 [file S0033291722000940sup001.docx]

# Supplementary materials

Sections

### Multiple regression

### Two Structural equation modelling approaches

### Assessing the characteristics of participants with differing levels of missingness

### Complete case analysis using latent basis growth curve modelling

1. Assessing the characteristics of participants across autism and each autistic trait
2. Reviewing the distribution of individual risk behaviours across autism and each autistic trait at age 16

## References

## List of tables and figures

- Table S1: Univariate and final adjusted multiple regression models for testing associations between autism-related exposures and MRBs in adolescence
- Table S2: Unconditional latent growth models depicting engagement in MRBs from ages ~12 to ~18 in ALSPAC participants with at least one MRBs score and complete MRB data only
- Table S3: Sociodemographic characteristics, exposures and outcomes of interest of ALSPAC participants in relation to level of missing MRB data
- Table S4: Sociodemographic characteristics, exposures and outcomes of interest of ALSPAC participants with at least 1 MRB score vs participants who did not have any recorded MRB scores
- Table S5: Conditional latent growth models depicting the effects of autism-related exposures on MRB from ages ~12 to ~18 in ALSPAC participants with complete MRB data only
- Table S6: Frequency distributions of sociodemographic characteristics, maternal psychopathology and MRBs across autism and each autistic trait
- Table S7: Frequency distributions of individual risk behaviours across autism and each autistic trait at age 16

### Multiple regression

Multiple models adjusting for sets of confounding factors were estimated: model 1 (m1): exposure and outcome; model 2 (m2): m1 + sex; model 3 (m3): m2 + all other covariates.

Results from models 1 and 3 are presented in Table S1. Adjusting for potential confounders did not substantially change the estimates. Social communication difficulties were associated with participation in a greater number of MRBs, on average, at every timepoint. Repetitive behaviours were also associated with greater MRBs participation at 12 and 14 years. Reduced sociability was associated with fewer MRBs at ~16 years and 18 years.

**Table S1: Univariate and adjusted multiple regression models for testing associations between autism-related exposures and MRBs in adolescence.**

|  |  | Model 1 |  |  | Model 3 |  |
| --- | --- | --- | --- | --- | --- | --- |
| Exposure | **N** | **β (95% CI)** | **p-value** | **N** | **β (95% CI)** | **p-value** |
| Autism cases | | |  |  |  |  |
| MRBs at ~12 | 3,938 | 0.15 (-0.24, 0.55) | 0.44 | 2,764 | 0.09 (-0.32, 0.49) | 0.67 |
| MRBs at ~14 | 4,097 | 0.03 (-0.31, 0.36) | 0.87 | 2,859 | 0.11 (-0.25, 0.47) | 0.47 |
| MRBs at ~16 | 2,674 | -0.31 (-0.99, 0.39) | 0.39 | 2,053 | -0.22 (0.47, 0.53) | 0.53 |
| MRBs at ~18 | 1,472 | -0.91 (-1.89, 0.06) | 0.07 | 1,157 | -0.80 (-1.85, 0.25) | 0.14 |
| Social communication and interaction difficulties (SCDC) | | | | | | |
| MRBs at ~12 | 3,614 | 0.30 (0.16, 0.44) | <0.001 | 2,641 | 0.28 (0.12, 0.45) | <0.001 |
| MRBs at ~14 | 3,688 | 0.31 (0.17, 0.45) | <0.001 | 2,708 | 0.31 (0.15, 0.47) | <0.001 |
| MRBs at ~16 | 2,416 | 0.68 (0.40, 0.97) | <0.001 | 1,941 | 0.69 (0.37, 1.01) | <0.001 |
| MRBs at ~18 | 1,358 | 0.78 (0.35, 1.21) | <0.001 | 1,111 | 0.90 (0.40, 1.40) | <0.001 |
| Pragmatic language difficulties | | |  |  |  |  |
| MRBs at ~12 | 3,695 | 0.20 (0.07, 0.33) | 0.01 | 2,672 | 0.12 (-0.04, 0.28) | 0.13 |
| MRBs at ~14 | 3,713 | 0.14 (0.01, 0.27) | 0.05 | 2,734 | 0.05 (-0.11, 0.21) | 0.55 |
| MRBs at ~16 | 2,455 | 0.30 (0.01, 0.59) | 0.04 | 1,964 | 0.22 (-0.10, 0.54) | 0.17 |
| MRBs at ~18 | 1,371 | -0.08 (-0.50, 0.33) | 0.70 | 1,112 | -0.20 (-0.69, 0.28) | 0.41 |
| Repetitive behaviours | | |  |  |  |  |
| MRBs at ~12 | 3,563 | 0.22 (0.06, 0.38) | 0.01 | 2,626 | 0.21 (0.02, 0.40) | 0.03 |
| MRBs at ~14 | 3,691 | 0.19 (0.03, 0.35) | 0.02 | 2,730 | 0.26 (0.07, 0.44) | 0.01 |
| MRBs at ~16 | 2,427 | 0.19 (-0.13, 0.50) | 0.24 | 1,959 | 0.15 (-0.19, 0.50) | 0.38 |
| MRBs at ~18 | 1,358 | -0.06 (-0.50, 0.38) | 0.78 | 1,112 | -0.09 (-0.58, 0.40) | 0.71 |
| Reduced sociability | | |  |  |  |  |
| MRBs at ~12 | 3,712 | 0.05 (-0.07, 0.17) | 0.40 | 2,724 | 0.10 (-0.03, 0.23) | 0.16 |
| MRBs at ~14 | 3,836 | -0.02 (-0.14, 0.10) | 0.73 | 2,816 | 0.10 (-0.12, 0.89) | 0.89 |
| MRBs at ~16 | 2,522 | -0.28 (-0.51, -0.05) | 0.02 | 2,027 | -0.30 (-0.55, -0.04) | 0.02 |
| MRBs at ~18 | 1,394 | -0.21 (-0.52, 0.10) | 0.18 | 1,146 | -0.33 (-0.68, 0.01) | 0.06 |

N – analytic sample size, β (95% CI) – beta coefficient representing a mean different point estimate. CI representing confidence intervals

### Two structural equation modelling approaches

We used Latent Growth Curve Modelling (LGCM) to estimate growth curves with the assumption of linear growth. We then used Latent Basis Growth Curve Modelling (LBGCM), which is a more flexible approach as growth patterns do not have to be pre-specified and thus, typically, the best-fitting non-linear trajectories are modelled^1^. The maximum likelihood with missing values estimation method (mlmv), also known as the full information maximum likelihood method (FIML), was used on data from participants who had at least 1 MRB score to include the maximum number of observations from participants in our analyses.

Initially, we fit unconditional latent growth models to model change in participants’ level of MRBs throughout adolescence without the influence of any exposures of interest. After comparing the two SEM techniques using postestimation statistics to assess goodness of model fit, we chose to use LBGC models as this technique produced the best fitting MRB trajectories. These analyses were repeated with complete cases.

As illustrated in Table S2, the average number of MRBs participated in at ~12 years of age was 1.57 (95% CI 1.54, 1.60) and the average increase in number of MRBs participated in with every passing year, was 0.28 (95% CI 0.27, 0.29) in the unconditional LGC models with at least one measurement of MRBs. The average number of MRBs participated in at 12 years of age was 1.69 (95% CI 1.66, 1.72) and the average increase in number of MRBs participated in with every passing year was 0.26 (95% CI 0.25, 0.28) in the unconditional LBGC models. After assessing the postestimation statistics from LGCM and LBGCM, the latter fit to the data better. After repeating the analyses with complete cases, there was only slight attenuation in MRBs participation at 12 years and across adolescence, with slightly wider 95% CIs and less precise goodness of fit statistics.

**Table S2:** **Unconditional latent growth models depicting engagement in MRBs from ages ~12 to ~18 in ALSPAC participants with at least one measurement of MRB (N=5,528) and complete MRB data only (N=706)**

|  | Latent growth curve modelling | Latent basis growth curve modelling |
| --- | --- | --- |
|  | **Β (95% CI)** | **Β (95% CI)** |
| Participants with at least one measurement of MRB | | |
| *n* | 5,528 | 5,528 |
| *Baseline MRB* | 1.57 (1.54, 1.60) | 1.69 (1.66, 1.72) |
| *Rate of change* | 0.28 (0.27, 0.29) | 0.26 (0.25, 0.28) |
| *Time @ wave 2* | 2* | 0.79 |
| *Time @ wave 3* | 4* | 4.91 |
| *Var(Intercept)* | 0.22 (0.17, 0.29) | 0.36 (0.31, 0.41) |
| *Var(Slope)* | 0.079 (0.069, 0.089) | 0.067 (0.059, 0.075) |
| *Covar(Int,Slope)* | 0.034 (0.014, 0.054) | 0.047 (0.032, 0.063) |
| *Corr(Int, Slope)* | 0.26 (0.07, 0.45) | 0.31 (0.19, 0.42) |
| *Residual variance* | 1.07 (1.02, 1.12) | 0.93 (0.89, 0.97) |
| *BIC* | 41352.5 | 40718.7 |
| *RMSEA* | 0.13 | 0.05 |
| *CFI* | 0.50 | 0.95 |
|  |  |  |
| Complete cases | | |
| *n* | 706 | 706 |
| *Baseline MRB* | 1.42 (1.35, 1.49) | 1.52 (1.45, 1.60) |
| *Rate of change* | 0.26 (0.23, 0.28) | 0.24 (0.21, 0.26) |
| *Time @ wave 2* | 2* | 0.85 |
| *Time @ wave 3* | 4* | 4.47 |
| *Var(Intercept)* | 0.14 (0.07, 0.30) | 0.24 (0.16, 0.35) |
| *Var(Slope)* | 0.060 (0.049, 0.073) | 0.056 (0.047, 0.068) |
| *Covar(Int,Slope)* | 0.034 (0.049, 0.061) | 0.038 (0.016, 0.061) |
| *Corr(Int, Slope)* | 0.37 (-0.04, 0.77) | 0.33 (0.09, 0.58) |
| *Residual variance* | 1.03 (0.95, 1.10) | 0.94 (0.87, 1.01) |
| *BIC* | 9405.0 | 9327.2 |
| *RMSEA* | 0.15 | 0.10 |
| *CFI* | 0.77 | 0.93 |

N – analytic sample size; β (95% CI) – beta coefficient representing a mean difference point estimate; CI representing confidence intervals; Var(Intercept) - variance of the intercept; Var(Slope) - variance of the slope; Covar(Int,Slope) – covariance of the intercept and slope; Corr(Int, Slope) – correlation between the intercept and slope; BIC - Bayesian information criterion, lower is preferred; RMSEA - Root Mean Square Error of Approximation, 0.1 is a marginal fit; CFI - comparative fit index CFI values range from 0 to 1, with larger values indicating better fit.

### Assessing the characteristics of participants with differing levels of missingness

To assess the differences in means and proportions of variables in participants with at least one MRBs score versus participants with no recorded MRBs scores, t-tests for continuous variables and chi-squared tests for categorical or binary variables were used. We also compared participants with no recorded MRBs scores to participants with one to three MRBs scores and participants with all MRBs scores. ANOVA and chi-squared tests for trend were performed.

There was greater prevalence of females and autistic traits and on average, IQ was higher and there were fewer cases of ID in the partially and none missing groups. SES was generally higher; prevalence of postnatal maternal depression was lower but maternal anxiety higher. Prevalence of maternal participation in risk behaviours was lower. The mean number of MRBs participated in at each time point was lower in the none missing group when compared to the partially missing group. For further details, please see Table S3.

**Table S3: Sociodemographic characteristics, exposures and outcomes of interest of ALSPAC participants in relation to level of missing MRB data**

|  | None missing (0) | Partially missing (1-3) | All missing (4) |  |
| --- | --- | --- | --- | --- |
|  | N = 706 | N =4822 | N= 8243 |  |
|  | **Mean (sd)** | **Mean (sd)** | **Mean (sd)** |  |
| MRBs at ~12 | **1.51 (1.01)** | **1.73 (1.13)** | **-** | <0.001 |
| MRBs at ~14 | **1.74 (1.19)** | **1.92 (1.18)** | - | <0.001 |
| MRBs at ~16 | 2.56 (1.68) | 3.03 (1.90) | - | <0.001 |
| MRBs at ~18 | 2.94 (1.93) | 3.11 (2.01) | - | 0.09 |
| IQ | 112.73 (14.89) | 105.54 (15.99) | 98.84 (16.25) | <0.001 |
| Maternal age | 30.61 (4.42) | 29.55 (4.51) | 27.60 (5.06) | <0.001 |
| Postnatal depression | 5.97 (4.60) | 6.59 (4.85) | 7.50 (5.23) | <0.001 |
|  |  |  |  |  |
|  | **Freq. (%)** | **Freq. (%)** | **Freq. (%)** |  |
| Sex - *Male* | 250 (35.41) | 2306 (47.82) | 4545 (55.14) | <0.001 |
| *Female* | 456 (64.59) | 2516 (52.18) | 3698 (44.86) |  |
| Autism cases *- Yes* | 7 (0.99) | 53 (1.10) | 95 (1.15) | 0.91 |
| *No* | 699 (99.01) | 4769 (98.90) | 8148 (98.85) |  |
| Social communication and interaction difficulties (SCDC) -*Yes* | 40 (5.67) | 360 (7.47) | 382 (4.63) | <0.001 |
| *No* | 633 (89.66) | 3842 (79.68) | 2733 (33.16) |  |
| Pragmatic language difficulties *- Yes* | 39 (5.52) | 404 (8.38) | 346 (4.20) | <0.001 |
| *No* | 644 (91.22) | 3869 (80.24) | 2384 (28.92) |  |
| Repetitive behaviours *-Yes* | 40 (5.67) | 258 (5.35) | 300 (3.64) | <0.001 |
| *No* | 633 (89.66) | 3946 (81.83) | 3296 (39.99) |  |
| Reduced sociability - *Yes* | 86 (12.18) | 467 (9.68) | 579 (7.02) | 0.04 |
| *No* | 598 (84.70) | 3969 (82.31) | 4219 (51.18) |  |
| Mum had anxiety or nerves* - Y*es* | 126 (17.85) | 829 (17.19) | 1001 (12.14) | 0.002 |
| *no* | 556 (78.75) | 3525 (73.10) | 3581 (43.44) |  |
| Maternal cannabis use* - Y*es* | 19 (2.69) | 156 (3.24) | 227 (2.75) | 0.001 |
| *no* | 663 (93.91) | 4198 (87.06) | 4355 (52.83) |  |
| Whether mum had major financial problems* - *Yes* | 88 (12.46) | 592 (12.28) | 805 (9.77) | <0.001 |
| *no* | 592 (83.85) | 3738 (77.52) | 3745 (45.43) |  |
| No. of cigarettes mother has smoked each day* - *none* | 608 (86.12) | 3523 (73.06) | 3107 (37.69) | <0.001 |
| *<10* | 30 (4.25) | 279 (5.79) | 370 (4.49) |  |
| *ten to nineteen* | 25 (3.54) | 304 (6.30) | 625 (7.58) |  |
| *20+* | 11 (1.56) | 135 (2.80) | 331 (4.02) |  |
| Quantity of alcohol mum drinks* - *don’t drink* | 60 (8.50) | 426 (8.83) | 628 (7.62) | <0.001 |
| *< once a week* | 291 (41.22) | 1949 (40.42) | 2122 (25.74) |  |
| *> once a week* | 244 (34.56) | 1494 (30.98) | 1334 (16.18) |  |
| *1-2 glasses everyday* | 76 (10.76) | 428 (8.88) | 411 (4.99) |  |
| *3-9 glasses everyday* | 9 (1.27) | 36 (0.75) | 41 (0.50) |  |
| Type of property *-Mortgaged/own* | 630 (89.24) | 3946 (81.83) | 4837 (58.68) | <0.001 |
| *Privately rent* | 39 (5.52) | 364 (7.55) | 972 (11.79) |  |
| *Sub rent* | 25 (3.54) | 380 (7.88) | 1648 (19.99) |  |
| Household income - *High* | 212 (30.03) | 951 (19.72) | 294 (3.57) | <0.001 |
| *Middle high* | 174 (24.65) | 880 (18.25) | 299 (3.63) |  |
| *Middle* | 139 (19.69) | 803 (16.65) | 319 (3.87) |  |
| *Middle low* | 99 (14.02) | 730 (15.14) | 317 (3.85) |  |
| *Low* | 48 (6.80) | 578 (11.99) | 348 (4.22) |  |
| Mother’s educational level *- < O-level* | 82 (11.61) | 955 (19.81) | 2639 (32.02) | <0.001 |
| *O-level* | 218 (30.88) | 1663 (34.49) | 2350 (28.51) |  |
| *> O-level* | 402 (56.94) | 2062 (42.76) | 1864 (22.61) |  |
| Parents' social class - *Professional* | 165 (23.37) | 695 (14.41) | 238 (2.89) | <0.001 |
| *Managerial and technical* | 321 (45.47) | 1876 (38.91) | 713 (8.65) |  |
| *Skilled non-manual* | 139 (19.69) | 989 (20.51) | 430 (5.22) |  |
| *Skilled manual, part or unskilled* | 60 (8.50) | 524 (10.87) | 331 (4.02) |  |

‘*since study child was 18 months old

MRBs – multiple risk behaviours; IQ – Intelligence Quotient as measured by the Wechsler Intelligence Scale for Children; SD – standard deviation

In the Table S4 below, results from comparing participants with at least one MRB score at any given timepoint to participants with no MRBs scores recorded during adolescence show these comparison groups generally differed with regards to socioeconomic measures, maternal participation in MRBs, maternal psychopathology and prevalence of autistic traits in the offspring. However, there were few differences in proportions of participants with diagnosed autism in the comparison groups. The differences between participants with no recorded MRBs scores, some MRBs score and all MRB scores follow a similar pattern.

**Table S4: Sociodemographic characteristics, exposures and outcomes of interest of ALSPAC participants with at least 1 MRB score vs participants who did not have any recorded MRB scores**

|  | At least one measurement of MRBs (n=5528) | All missing (n=8243) | p-value^1^ |
| --- | --- | --- | --- |
|  | **mean (SD)** | **mean (SD)** |  |
| MRBs at ~12 | 1.69 (1.11) | **-** | - |
| MRBs at ~14 | 1.89 (1.17) | **-** | - |
| MRBs at ~16 | 2.91 (1.86) | **-** | - |
| MRBs at ~18 | 3.03 (1.97) | **-** | - |
| IQ | 105.54 (16.03) | 98.84 (16.25) | <0.001 |
| Maternal age | 29.69 (4.51) | 27.6 (5.07) | <0.001 |
| Maternal depression score* | 6.51 (4.82) | 7.50 (5.23) | <0.001 |
|  |  |  |  |
|  | **Freq. (%)** | **Freq. (%)** |  |
| Sex - *male* | 2556 (46.24) | 4545 (55.14) | <0.001 |
| *Female* | 2972 (53.76) | 3698 (44.86) |  |
| Autism cases- *Yes* | 60 (1.09) | 95 (1.15) | 0.71 |
| *no* | 5468 (98.91) | 8148 (98.85) |  |
| Social communication and interaction  difficulties (SCDC) - *Yes* | 400 (7.24) | 382 (4.63) | <0.001 |
| *no* | 4475 (80.95) | 2733 (33.16) |  |
| Pragmatic language difficulties *-Yes* | 443 (8.01) | 346 (4.20) | <0.001 |
| *no* | 4513 (81.64) | 2384 (28.92) |  |
| Repetitive behaviours *- Yes* | 298 (5.39) | 300 (3.64) | <0.001 |
| *no* | 4579 (82.83) | 3296 (39.99) |  |
| Reduced sociability *- Yes* | 553 (10.00) | 579 (7.02) | 0.05 |
| *no* | 4567 (82.62) | 4219 (51.18) |  |
| Mum had anxiety or nerves* - *Yes* | 955 (17.28) | 1001 (12.14) | <0.001 |
| *no* | 4081 (73.82) | 3581 (43.44) |  |
| Maternal cannabis use* - *Yes* | 175 (3.17) | 227 (2.75) | <0.001 |
| *no* | 4861 (87.93) | 4355 (52.83) |  |
| Whether mum had major financial problems* - *Yes* | 680 (12.30) | 805 (9.77) | <0.001 |
| *no* | 4330 (78.33) | 3745 (45.43) |  |
| No. of cigarettes mother has smoked each day* - *none* | 4131 (74.73) | 3107 (37.69) | <0.001 |
| *<10* | 309 (5.59) | 370 (4.49) |  |
| *10-19* | 329 (5.95) | 625 (7.58) |  |
| *20+* | 146 (2.64) | 331 (4.02) |  |
| Quantity of alcohol mum drinks* -*don’t drink* | 486 (8.79) | 628 (7.62) | <0.001 |
| *< once a week* | 2240 (40.52) | 2122 (25.74) |  |
| *> once a week* | 1738 (31.44) | 1334 (16.18) |  |
| *1-2 glasses everyday* | 504 (9.12) | 411 (4.99) |  |
| *3-9 glasses everyday* | 45 (0.81) | 41 (0.50) |  |
| Type of property *-Mortgaged/own* | 4576 (82.78) | 4837 (58.68) | <0.001 |
| *Privately rent* | 403 (7.29) | 972 (11.79) |  |
| *Sub rent* | 405 (7.33) | 1648 (19.99) |  |
| Household income *- High* | 1163 (21.04) | 294 (3.57) | <0.001 |
| *Middle high* | 1054 (19.07) | 299 (3.63) |  |
| *Middle* | 942 (17.04) | 319 (3.87) |  |
| *Middle low* | 829 (15.00) | 317 (3.85) |  |
| *Low* | 626 (11.32) | 348 (4.22) |  |
| Mother’s educational level *-< O-level* | 1037 (18.76) | 2639 (32.02) | <0.001 |
| *O-level* | 1881 (34.03) | 2350 (28.51) |  |
| *> O-level* | 2464 (44.57) | 1864 (22.61) |  |
| Parents' social class *-Professional* | 860 (15.56) | 238 (2.89) | <0.001 |
| *Managerial and technical* | 2197 (39.74) | 713 (8.65) |  |
| *Skilled non-manual* | 1128 (20.41) | 430 (5.22) |  |
| *Skilled manual, part or unskilled* | 584 (10.56) | 331 (4.02) |  |

### ‘*Since the study child was 18 months old

### ^1^ from hypothesis testing to assess differences between participants with at least one measurement of MRBs and those with none.

MRBs – multiple risk behaviours; IQ – Intelligence Quotient as measured by the Wechsler Intelligence Scale for Children; SD – standard deviation

### Complete case analysis using latent basis growth curve modelling

**Table S5: Unadjusted associations between autism-related exposures and MRB from ages ~12 to ~18 in ALSPAC participants with complete MRB data only (N=673-706)**

|  | *Effect of exposure on baseline MRB* | | *Effect of exposure on rate of change* | |
| --- | --- | --- | --- | --- |
|  | **Β (95% CI)** | **p-value** | **Β (95% CI)** | **p-value** |
| Autism cases | -0.19 (-0.85, 0.46) | 0.57 | -0.20 (-0.43, 0.03) | 0.08 |
| SCDC | 0.17 (-0.11, 0.46) | 0.22 | 0.13 (0.03, 0.23) | 0.01 |
| Pragmatic language difficulties | 0.18 (-0.10, 0.47) | 0.20 | -0.07 (-0.17, 0.03) | 0.16 |
| Repetitive behaviours | 0.07 (-0.22, 0.35) | 0.63 | -0.03 (-0.13, 0.07) | 0.56 |
| Reduced sociability | 0.02 (-0.18, 0.22) | 0.84 | -0.05 (-0.12, 0.02) | 0.14 |

As illustrated in Table S5, there was only evidence for an above average uptake of number of MRBs with each passing year during adolescence in participants with social communication difficulties compared to typically developing participants in the complete case analysis.

1. Assessing the characteristics of participants across autism and each autistic trait

**Table S6: Frequency distributions of sociodemographic characteristics, maternal psychopathology and MRBs across autism and each autistic trait**

|  | N (%) with autism or autistic traits | | | | |
| --- | --- | --- | --- | --- | --- |
|  | **Autism (yes)** | **SCDC (yes)** | **Pragmatic Language difficulties (yes)** | **Repetitive**  **Behaviours (yes)** | **Sociability (yes)** |
| Sex (male) | 45 (1.76) | 239 (10.45) | 271 (11.62) | 171 (7.47) | 289 (12.11) |
| Mum anxiety *(yes)* | 11 (1.15) | 94 (10.98) | 95 (10.92) | 75 (8.54) | 87 (9.35) |
| Mum cannabis *(yes)* | 0 (0) | 11 (7.14) | 16 (10.26) | 13 (8.39) | 13 (7.78) |
| Mum financial problems *(yes)* | 7 (1.03) | 62 (10.44) | 59 (9.72) | 56 (9.20) | 61 (9.24) |
| Mum *smoking (yes)* | 6 (0.95) | 68 (9.92) | 69 (9.97) | 48 (6.81) | 67 (8.93) |
| Mum alcohol *(daily)* | 8 (1.48) | 44 (8.54) | 47 (9.13) | 24 (4.69) | 55 (10.36) |
| *Moderate* | 42 (1.07) | 285 (7.86) | 319 (8.68) | 220 (5.98) | 414 (10.66) |
| *Doesn’t drink* | 10 (2.06) | 44 (10.30) | 42 (9.68) | 37 (8.41) | 60 (12.99) |
| Housing tenure *(mortgaged/owned)* | 56 (1.22) | 327 (7.95) | 364 (8.75) | 241 (5.86) | 467 (10.85) |
| *Private rental* | 4 (0.99) | 29 (8.55) | 27 (7.52) | 27 (7.76) | 45 (12.47) |
| *Subsidised rental* | 0 (0) | 36 (11.08) | 44 (13.29) | 26 (8.00) | 37 (10.39) |
| Household income (high) | 18 (1.55) | 87 (7.91) | 84 (7.62) | 60 (5.48) | 88 (7.74) |
| *Middle high* | 13 (1.23) | 66 (6.80) | 77 (7.84) | 53 (5.41) | 102 (9.96) |
| *Middle* | 9 (0.96) | 58 (6.77) | 72 (8.37) | 45 (5.20) | 109 (11.98) |
| *Middle low* | 9 (1.09) | 75 (10.03) | 77 (10.17) | 49 (6.33) | 98 (12.19) |
| *Low* | 9 (1.44) | 50 (9.36) | 63 (11.60) | 47 (8.53) | 82 (13.76) |
| Mum education level *(>O-level)* | 37 (1.50) | 179 (7.87) | 188 (8.13) | 134 (5.92) | 230 (9.80) |
| *O-level* | 12 (0.64) | 117 (7.03) | 142 (8.39) | 96 (5.68) | 190 (10.78) |
| *<O-level* | 11 (1.06) | 98 (11.56) | 101 (11.73) | 65 (7.61) | 125 (13.44) |
| Parental social class *(professional)* | 11 (1.28) | 52 (6.49) | 67 (8.22) | 51 (6.39) | 79 (9.65) |
| *Managerial/technical* | 37 (1.68) | 171 (8.50) | 172 (8.40) | 112 (5.58) | 221 (10.62) |
| *Skilled non-manual* | 4 (0.35) | 78 (7.82) | 97 (9.69) | 65 (6.42) | 123 (11.63) |
| *Skilled manual, part or unskilled* | 5 (0.86) | 45 (9.22) | 41 (8.40) | 29 (5.89) | 61 (11.42) |
| Maternal depression  *(median, IQR)* | 6.5 (3,11) | 8 (4,12) | 7 (4, 11) | 7 (4,11) | 6 (3,10) |
| Maternal age *(mean, sd)* | 30.39 (3.98) | 29.81 (4.59) | 29.75 (4.44) | 29.5 (4.44) | 29.74 (4.27) |
| IQ *(mean, SD)* | 100.41 (19.82) | 102.17 (19.13) | 97.93 (16.82) | 103.63 (17.60) | 105.55 (15.27) |
| MRBs at ~ 12 yo *(median, IQR)* | 2 (1,2) | 2 (1, 3) | 2 (1,3) | 2 (1,3) | 2 (1,2) |
| MRBs at ~ 14 yo *(median, IQR)* | 2 (1, 2.5) | 2 (1,3) | 2 (1,3) | 2 (1,3) | 2 (1,2) |
| MRBs at ~ 16 yo *(median, IQR)* | 2 (1, 3) | 3 (2, 5) | 3 (2,4) | 3 (2, 4) | 2 (1,4) |
| MRBs at ~ 18 yo *(median, IQR)* | 2 (1,3) | 4 (2, 5) | 3 (2, 4) | 2 (2,4) | 3 (2,4) |

N (%) - number of participants in this category, column percentages; SCDC - social communication and interaction difficulties as measured by the social communication difficulties checklist; SD - standard deviation; IQR - Interquartile range

In Table S6, results indicate that there are small numbers for analysis with autism diagnosis as an exposure and there was more loss to follow up in the group with higher levels of repetitive behaviours, given that approximately the 10% most affected by the trait were selected and that fewer participants were distributed across categories of variables. There is some indication of lower levels of home ownership and lower maternal educational levels.

1. Reviewing the distribution of individual risk behaviours across autism and each autistic trait at age 16

**Table S7: Frequency distributions of individual risk behaviours across autism and each autistic trait at age 16**

|  | N (%) of engagement in each risk behaviour | | | | | |
| --- | --- | --- | --- | --- | --- | --- |
|  | **Autism (yes)** | **SCDC (yes)** | **Pragmatic language difficulties (yes)** | **Repetitive**  **Behaviours (yes)** | **Sociability (yes)** |  |
| Car passenger risk *(yes)* | 8 (0.89)* | 76 (9.13)* | 58 (6.88) | 49 (5.81) | 96 (10.80) |  |
| Scooter risk *(yes)* | 5 (0.99) | 42 (8.81) | 37 (7.79) | 30 (6.33) | 47 (9.40) |  |
| Cycle helmet use *(yes)* | 15 (2.01) | 69 (9.7)* | 61 (8.51) | 38 (5.42) | 79 (10.75) |  |
| Illicit drug use *(yes)* | <5 | 16 (12.21)* | 12 (9.45) | 7 (5.43) | 8 (6.06)* |  |
| Cannabis use *(yes)* | 6 (1.85) | 36 (12.04)* | 21 (7.02) | 28 (9.21)* | 32 (10.09) |  |
| Smoking *(yes)* | 5 (1.35) | 30 (8.72) | 24 (7) | 25 (7.31) | 29 (8.03)* |  |
| Hazardous alcohol consumption *(yes)* | 10 (0.92)* | 82 (8.13) | 73 (7.14) | 52 (5.08) | 99 (9.37)* |  |
| Unprotected sex *(yes)* | 0 | 5 (10.64) | <5 | <5 | <5 |  |
| Risky sexual behaviour *(yes)* | <5 | 10 (12.5) | <5 | 5 (5.95) | 7 (7.95) |  |
| Self-harm *(yes)* | 8 (1.33) | 53 (9.58)* | 47 (8.39) | 37 (6.49) | 57 (9.74) |  |
| Anti-social/criminal behaviour *(yes)* | 18 (1.09) | 162 (10.58)* | 135 (8.78) | 105 (6.82)* | 179 (11.08) |  |
| Excessive TV watching *(yes)* | 11 (1.79) | 49 (8.63) | 60 (10.51)* | 36 (6.33) | 70 (11.69) |  |
| Physical inactivity *(yes)* | 44 (1.46) | 226 (8.12) | 238 (8.45) | 172 (6.11) | 335 (11.38) |  |

N (%) - number of participants in this category, column percentages; SCDC - social communication and interaction difficulties as measured by the social communication difficulties checklist

Following chi-squared testing, results with an asterisk were <0.05 or very close to it.

To briefly summarise Table S7, there were differences in car passenger risk and hazardous drinking behaviours when comparing those with autism cases and non-autistic cases. There were most differences between those with high levels of social communication difficulties and those without, namely in car passenger risk, cycle helmet use, illicit drug use, cannabis use, self-harm and anti-social/criminal behaviours. There were fewer differences between those with high levels of autistic traits and those with less or no difficulties in these areas.

## References

1. Grimm KJ, Ram N, Hamagami F. Nonlinear Growth Curves in Developmental Research. 2011;82(5):1357-1371.
